# Oral hygiene in intensive care unit patients with photodynamic therapy: study protocol for randomised controlled trial

**DOI:** 10.1186/s13063-017-2133-y

**Published:** 2017-08-22

**Authors:** Gabriela Alves Da Collina, Anna Carolina Ratto Tempestini-Horliana, Daniela de Fátima Teixeira da Silva, Priscila Larcher Longo, Maria Luisa Faria Makabe, Christiane Pavani

**Affiliations:** 1 Biophotonics Applied to Health Sciences Postgraduation Program, Universidade Nove de Julho – UNINOVE, R. Vergueiro, 235/249, CEP 01504-001 São Paulo, Brazil; 2 School of Dentistry, Universidade Nove de Julho – UNINOVE, São Paulo, Brazil

**Keywords:** Nosocomial infections, Intensive care units, Photodynamic therapy, Methylene blue mouthwash, Dentistry

## Abstract

**Background:**

In intensive care units (ICUs), nosocomial infections are prevalent conditions and they have been related to high mortality indexes. Some studies have suggested that inefficient oral hygiene and ventilator-associated pneumonia (VAP) are related. Nowadays, in the Brazilian public health system there is no well-defined protocol for oral hygiene in an ICU. Due to the drawbacks of the use of antibiotics, photodynamic therapy (PDT) has emerged as an interesting technique in order to reduce antimicrobial-resistant pathogens. Methylene blue (MB) is the most common chemical agent for PDT in Brazil. However, new formulations for improved effectiveness are still lacking. The objective of this study is to evaluate the use of an MB mouthwash as an effective oral-hygiene procedure in an ICU and to show that oral hygiene using PDT with MB mouthwash may reduce VAP frequency to rates similar to, or higher than, chlorhexidine.

**Methods:**

Phase 1 will evaluate the most effective cleaning procedure, while phase 2 will correlate oral hygiene to VAP incidence. At the start of phase 1, the ICU patients will be randomly allocated into three different groups (10 patients/group): the efficacy of chlorhexidine, classical MB-PDT, and mouthwash MB-PDT will all be measured for the quantification of viable bacteria, both pre- and post-treatment, by a Reverse Transcription Polymerase Chain Reaction (RT-PCR). In phase 2, the most effective procedure found in phase 1 and a mechanical cleaning with filtered water will be carried out daily, once a day, over 5 days, with a total of 52 ICU patients randomly allocated into the two groups. The clinical records will be evaluated in order to find any pneumonic diagnoses.

**Discussion:**

Since a variety of bacterial species are related to VAP, a universal primer for bacteria will be used in order to quantify the total bacteria count in the participants’ samples. In order to quantify only the living bacteria before DNA extraction, the samples will be treated with propidium monoazide. This will infiltrate the dead bacteria and will intercalate the DNA bases, avoiding their DNA amplification. This will be the first trial to evaluate MB-PDT in a mouthwash formula that can increase the effectiveness due to the control of MB aggregation. The results of this study will be able to generate an easy and low-cost protocol to be used in an ICU for the Brazilian public health system.

**Trial registration:**

This protocol was approved by the Research Ethics Committee of the Conjunto Hospitalar do Mandaqui (1.317.834, CAAE: 49273515.9.3001.5551) and it was registered in Registro Brasileiro de Ensaios Clínicos (ReBEC number: RBR-94bvrc;). First received: 12 July 2015; 1st version 6 June 2016. Data will be published in a peer-reviewed journal.

**Electronic supplementary material:**

The online version of this article (doi:10.1186/s13063-017-2133-y) contains supplementary material, which is available to authorized users.

## Background

It has been proposed that the oral cavity may be the dissemination focus of potentially pathogenic organisms to other body locations [1–4]. In intensive care units (ICU), infectious diseases are prevalent and they are responsible for the high mortality index of inpatients. The lung is the most affected organ mainly due to mechanical ventilation [5]. Ventilator-associated pneumonia (VAP) is defined as pneumonia in a patient receiving mechanical ventilation for at least 48 h that is not pre-existent at the moment of intubation [6]. The most prevalent pathogenic agents related to pneumonia are *Pseudomonas aeruginosa* 30.1%), *Staphylococcus aureus* (19.6%), *Acinetobacter* spp. (13.0%), *Klebsiella* spp. (9.5%) and *Enterobacter* spp. (8.4%) [7]. Both VAP control and prevention are important in an ICU since this can reduce hospitalisation time and costs [8].

During a patient’s stay in an ICU, oral biofilms and tongue coating increase simultaneously. Due to the orotracheal intubation, a patient’s mouth is open during the entire period, increasing bacterial plaque formation and reducing saliva flow. Consequently, this reduces the innate defence system and increases the risk of VAP [5]. Therefore, oral hygiene in an ICU is essential when implementing VAP-prevention strategies. However, developing these new strategies is a challenge, since many substances that have been suggested are associated with bacterial resistance; for example, chlorhexidine [9, 10].

In this context, photodynamic therapy (PDT) has shown effective results in killing microorganisms by a suitable combination of a photosensitiser dye, light and oxygen [11–19]. PDT’s advantages over other antimicrobial treatments are an efficacy that is independent of microorganism resistance and its broad spectrum of action. This is because a photosensitiser can act on bacteria, fungi, viruses, parasites, etc. [11, 16, 20–24]. This technique is minimally invasive, with no collateral effects to the host and it can be considered to be economically viable. The topical application of a photosensitiser is interesting since it is in direct contact with the microorganisms and it causes low collateral effects in the patient [12, 25]. In PDT treatment, after light absorption by the photosensitiser and the generation of excited states, two main photochemical mechanisms of damage to biomolecules are involved: in Type-I reactions, due to electronic transfer, the photosensitiser in its triplet excited state generates reactive oxygen species (mainly radicals); in Type-II reactions, there is the generation of singlet oxygen after energy transfer (^1^O_2_) [19]. The reaction type can affect the therapeutic efficacy since the reactivity to the targets can differ considerably [19, 26]. However, there is a lack of information regarding the relationships between the photochemical mechanisms and the inactivation efficiencies in different microorganisms. While some articles have affirmed that singlet oxygen is the main oxidising agent [27], others have suggested that reactive oxygen species (mainly radicals) are more effective at killing Gram-negative bacteria and that singlet oxygen is more effective at killing Gram-positive bacteria [21, 28].

Since microorganism membranes are negatively charged, the photosensitiser must be positively charged in order to efficiently penetrate these membranes [29]. Therefore, methylene blue (MB) has been broadly used due to its positive charges, its effectiveness and its low cost [30, 31]. Besides, MB has been frequently used in medicine, since its safety and toxicity are well known and the concentrations used in PDT are lower than those used for other medical purposes [32–35]. Methylene blue has been shown to be effective against bacteria, viruses, parasites and fungi [22, 36–39]. However, the efficacy is dependent on the microorganisms (structure, thickness and the composition of the biofilm matrix, efflux pumps, etc.) and on the treatment parameters such as MB concentrations and irradiance [29, 40–42]. Besides, the MB vehicle can affect the efficacy of PDT, although this is rarely considered. Depending upon the physicochemical environment, MB may aggregate and this feature modulates the photochemical reactions [43, 44]. While MB monomers generate singlet oxygen, MB dimers induce the production of other reactive oxygen species, mainly radicals [43]. Some studies have shown ways to control MB aggregation and these strategies can be used in order to develop specific MB formulations in which monomers or dimers are preferred [43, 45–49]. For example, Nunez et al. showed a greater ionic strength in physiological media and that saliva induced the formation of MB dimers, reducing their efficacy [46]. On the other hand, urea drives monomer formation and enhances MB PDT effectiveness [45]. Based on this information, we have designed a mouthwash formula containing MB with the aim of stabilizing MB monomers and improving the PDT outcomes. The mouthwash formula is under a patent requirement.

Regarding these circumstances, the objective of this study will be to compare classical PDT with MB in water to a mouthwash formula for oral-hygiene procedures for ICU patients. The outcome to be measured will be the oral bacteria count, measured in pre- and post-intervention samples, with three experimental groups, by using a Reverse Transcription Polymerase Chain Reaction (RT-PCR) in samples pre-treated with propidium monoazide (PMA). Finally, it is also an objective of this work to show that oral hygiene using PDT with MB mouthwash may reduce VAP frequency to rates similar to, or higher than, chlorhexidine. The outcome will be to constitute a VAP diagnosis by checking the patients’ medical cards, after 5 days of daily oral-hygiene procedures. The results of this protocol will yield interesting tools that will raise attention to ICU oral-hygiene procedures. Additionally, it will establish an easy and low-cost oral-hygiene protocol to be used in an ICU for the Brazilian public health system, as well as being an important observation regarding the MB vehicle in PDT clinical protocols.

## Methods

### Study design

This study will be a randomised controlled, double-blind, single-centre, clinical trial. This study protocol was written based upon the Standard Protocol Items: Recommendations for Interventional Trials (SPIRIT) guidelines (see Additional file 1) and will be carried out at an ICU of a hospital in the Brazilian public health system in the city of São Paulo, Brazil (Hospital Assembly of Mandaqui). Since the patients will be unconscious during the procedures, the oral-hygiene procedures will be conducted by researcher GAC, together with a sample collection by researcher MLFM, both authors of this protocol study. This trial can be considered to be double blind. For better understanding, this study will be described in two phases, although two different studies will be performed, nested into each other. In phase 1, PDT with an MB mouthwash will be compared to PDT with MB in water, using chlorhexidine as positive control for oral hygiene. In phase 2, the frequencies of VAP occurrence will be measured in the two groups: (1) the most effective procedures found in phase 1 group and (2) the mechanical cleaning with filtered water group.

### Sample size calculation

First, a published paper using the same method of sample analyses (PCR + PMA pre-treatment) is to be used to determine the effect size (*Δ*). The largest and the smallest mean values (1.69 and 0.27, respectively), as well as the standard deviation (*σ* = 0.36) of the PCR quantification of living bacteria using PMA were taken from the Àlvarez article, 2013 [50]. The *n* value is to be the number of treatment groups, i.e., 3:

$$ \varDelta =\frac{Largest- smallest}{{\left(\frac{\sigma }{\sqrt{n}}\right)}^2}=\frac{1.69-0.27}{{\left(\frac{0.36}{\sqrt{3}}\right)}^2}=32.8 $$

G*Power software (version 3.1.9.2, Dusseldorf, Germany) was used to calculate the sample size. Using the calculated effect size, *F* tests will be chosen for the repeated measures and for the within-between interactions. Three groups will be studied and each group will be measured before and after the oral-hygiene procedures (i.e. three groups and two measurements). The sample size was determined by setting a two-sided error at 5% and the test power at 95% of the test. According to the calculations, a sample of six patients per group will be necessary in order to detect the differences in oral hygiene in phase 1. When considering this result and the possibility of difficulties in the sample processing, phase 1 will be conducted with 10 patients per group.

The sample size calculation for phase 2 was based on the percentages of patients with VAP before and after the implementation of oral-dental care, as published by Garcia et al. [8]. Using G*Power software, when choosing the Exact Family Test and Fisher’s Exact Test as statistical tests (inequality, two independent groups) and by inserting the proportions of 0.086 and 0.041 [8], the sample size was found to be 52, i.e. 26 patients per group. It is important to highlight that at this phase, a 5-day protocol will be evaluated. Due to the condition of the patients, some of them may recover and the mechanical ventilation will be removed, while some may get worst or even die. Due to these potential dropout patients, a sample size of 70 patients (35 per group) will be used.

### Sample size

In phase 1, 30 patients will be divided into three experimental groups (10 patients per group: chlorhexidine, classical MB-PDT and mouthwash MB-PDT). In phase 2, a total of 70 patients will be selected, 35 patients in each experimental group.

### Inclusion/exclusion criteria

The inclusion criteria are: ICU patients with 0- to 24-h orotracheal intubation; patients whose family signs the Consent Form; both genders, aged above 18 years old; and edentulous. The exclusion criteria are: reintubation; readmission in the ICU; smokers and ex-smokers for less than 5 years.

### Randomisation

Randomisation will be conducted by a researcher not involved in the treatment of the patients (ACRTH, author of this study protocol) when using Excel 2013 (Microsoft, Redmond, WA, USA). Opaque envelopes with sequential numbers will be used and a paper will be received containing the experimental group’s information according to the random draw. The envelopes will be sealed and stored in a safe place. ACRTH will be responsible for the storage and the confidentiality of the research envelopes. Immediately before the treatments, GAC will receive the envelopes from ACRTH, open them (the first in sequence) and accomplish the indicated procedures.

#### Interventions in phase 1

Within 24 h of the start of the mechanical ventilation and after checking the patients’ eligibility and obtaining the Consent Form signed by the person responsible for the patient, the intervention will be conducted. First, a patient’s sample will be collected by MLFM. Following this, the oral-hygiene procedure will be performed by GAC with a gauze (7.5 cm × 7.5 cm size and sterile) soaked in a solution that will be determined by randomisation: (1) positive control group: chlorhexidine 0.12%, (2) MB 0.05% aqueous solution, (3) MB 0.05% blue mouthwash (this will be prepared in a pharmacy – the formula is under patent requirement). Oral hygiene will be initiated from the right upper arcade (the labial surface), passing to the left arcade (no return). Then, the palatal hygiene will be conducted from left to right. When the upper arch has been completed, the left buccal mucosa will be hygienised, then the lower arch moving from left to right. At conclusion, the mouth floor, the tongue and the lips will be hygienised (due to the dryness caused by the salivary reduction). After the mechanical cleaning and the active application of mouthwash, 5 min of incubation will be necessary before starting the light exposures. The light system will be a Lineaxul Bucal (Cosmedical, Brazil) which is a light-emitting diode (LED) device emitting at the red region of the spectra (660 nm). The device is a flexible lollipop-shaped instrument composed of six LED points on both sides, allowing for simultaneous illumination. Three devices will be used simultaneously: one above the tongue, promoting the illumination of the palate region and two others positioned on the buccal mucosa (right and left). Using this composition, the oral cavity will be illuminated as a whole for 15 min. Another sample will be collected by sterile swabs soaked in a physiological solution (sodium chloride 0.9%), 30 min after the procedure in the retromolar trigone region. These swabs will be stored in sterile tubes containing Tris-EDTA buffer (Tris 10 mM, pH 8, EDTA 1 mM) and will be identified with a patient number (received at randomisation) and a pre- and post-treatment label (T_0_ – before treatment; T_1_ – after treatment) [35]. Any modifications in the protocol will be informed in the results paper.

### Sample processing

**Fig. 1:**
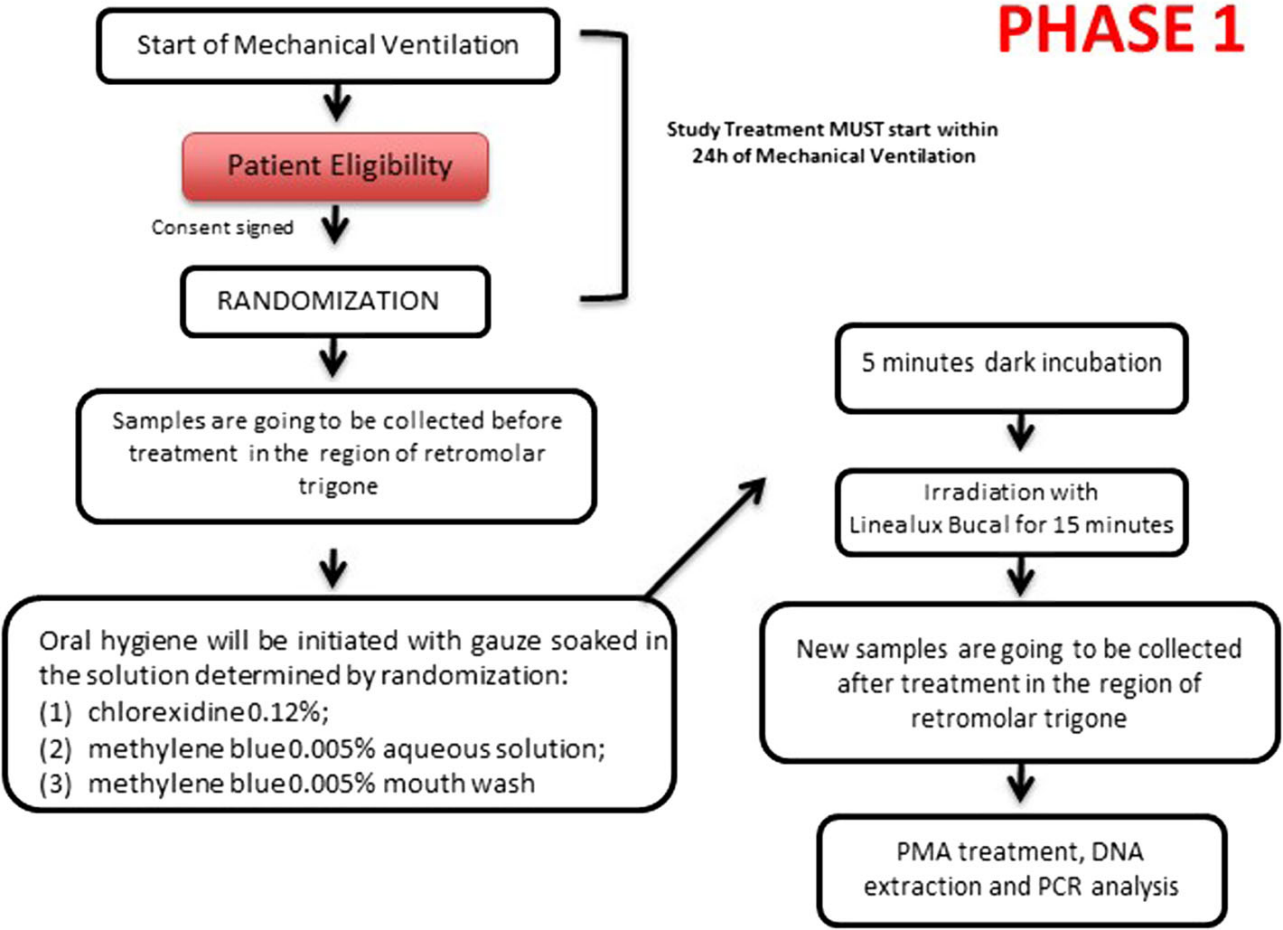
Phase-1 flow diagram

The samples will be treated with PMA 100 μmol/L for 10 min in the dark followed by 10 min of light exposure (LED device emitting at 470 nm, 3.2 MW/cm^2^) [50]. After the photoactivation, the cells will be centrifuged (8000 g, 10 min) and the deoxyribonucleic acid (DNA) extractions will be carried out using the manufacturer’s instructions (Master Pure DNA Extraction Kit – Epicentre Technologies Corp., Chicago, IL, USA). The total bacteria analyses will be carried out by real-time PCR (StepOnePlus Real-Time PCR System, Applied Biosystems^TM^, Waltham, MA, USA) and the products will be detected by fluorescence when employing a Quantimix Easy SYG Kit (Biotools Biotechnological & Medical Laboratories SA, Madrid, Spain) using the manufacturer’s protocol. During the PCR, a universal primer will be used in order to analyse the total bacteria count before and after the treatments. The reactions will start with a denaturation (95 °C for 2 min), 36 cycles of 30 s at 94 °C, 1 min at 55 °C and 2 min at 72 °C, with a final extension of 10 min at 72 °C. After each cycle, the fluorescence will be detected and a graph will be presented. All of the samples will be analysed twice and each dilution in a standard curve will be performed in triplicate. Figure 1 will describe a complete flow diagram for phase 1 from the patients’ enrolment, the interventions, the sample collections, the processing and the analyses.

#### Interventions in phase 2

**Fig. 2:**
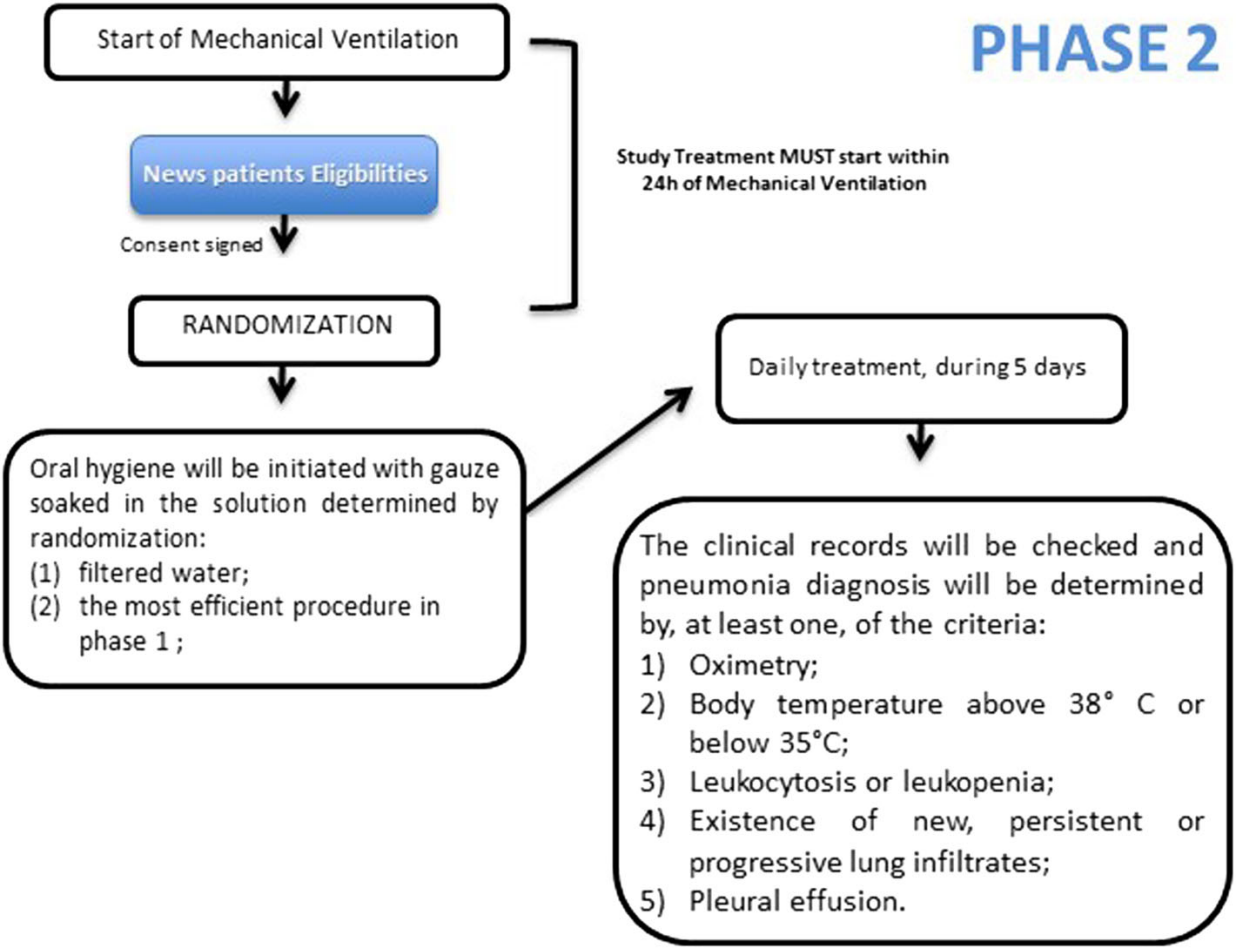
Phase-2 flow diagram

Based upon the results generated in phase 1, a new group of patients will comprise phase 2 of this study. Here, the oral-hygiene procedures described above will be performed in two groups of patients: (1) the most effective procedures in phase 1 group and (2) the mechanical cleaning with filtered water group. These procedures will be performed daily, over 5 days, when the outcomes will be evaluated. The clinical records will be checked and the pneumonia diagnoses will be determined by at least one of the following criteria: oximetry; body temperatures above 38 °C or below 35 °C; a leukocytosis or a leukopenia (leukocytes) count in the peripheral blood that is below 4000/mm^3^ or above 11,000/mm^3^; the existence of new, persistent or progressive lung infiltrates, or pleural effusion [51–54]. If any of the patients present an adverse reaction, they will be removed from the study and this will be reported in the published results paper. All of the personal information of the patients will be kept confidential. Figure 2 will describe the complete flow diagram for phase 2.

### Outcomes

Phase 1: the main outcome will be the efficacy of the oral-hygiene procedures, evaluated by the total bacteria count, before and after the treatments (three groups and two time points). A secondary outcome that can be measured is the change in the pathogenic bacteria count that is related to VAP after the oral-hygiene procedures.

Phase 2: the main outcome will be the rate of VAP, evaluated by checking the medical cards, 5 days after the daily oral-hygiene procedures.

**Fig. 3:**
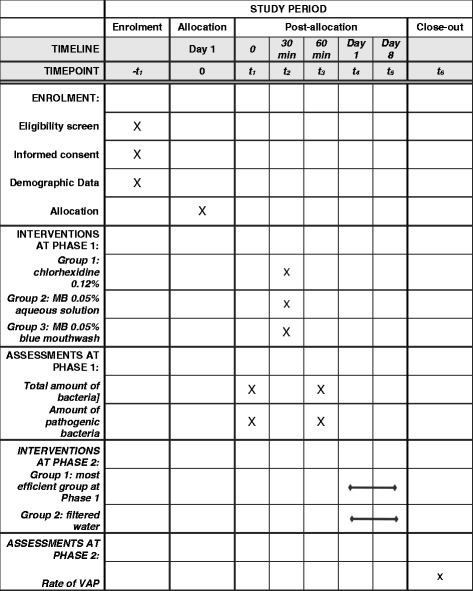
Standard Protocol Items: Recommendations for Interventional Trials (SPIRIT) Figure: schedule of enrolment, interventions and assessments

The detailed schedule for the study is presented in the SPIRIT figure (Fig. 3).

### Data analysis plan

The studied parameters in this work will aim to follow a Gaussian (normal) distribution and they will be verified by the Shapiro-Wilk test. Where the data are not parametric, they will be transformed by using a logarithm or any other math transformation into normally distributed data. In this manner, parametric methods will be used in order to detect the differences among the groups. In order to show the actual distribution of the measured data, a graph based on the mean values and standard deviations will be used. For the data analyses, SPSS Statistics^TM^ Version 22 Software (IBM, Armonk, NY, USA) will be used. The statistical analyses will be realised by using analysis of variance (ANOVA): this will include the repeated measures as well as the within- and between-group interactions since three groups will be studied and each group will be measured before and after the oral-hygiene procedures. The frequencies of VAP occurrence will be measured in the two groups: (1) the most effective procedures found in phase 1 group and (2) the mechanical cleaning with filtered water group. The chi-square test will be used to verify the difference between the observed frequencies. The significance level for all of the tests will be *p* < 0.05. The results of this study will be published in an international journal.

## Discussion

Some studies have suggested that inefficient oral hygiene and VAP are related [6, 8, 55–58]. Hua et al. recently concluded in a systematic review that oral hygiene reduces the risk of developing VAP from 25% to about 19%. Nonetheless, no differences in the outcomes of mortality, the duration of the mechanical ventilations, or the duration of ICU stay were detected [59]. This protocol will aim to specifically verify the efficacy of PDT in the context of an ICU, when considering that a more effective oral-hygiene protocol can reduce VAP incidence. This can help in a patient’s recovery, reducing their ICU stay, together with the treatment costs to the Brazilian public health system. Besides, it is known that critically ill patients, such as ICU inpatients, present a different oral microbiome, mostly composed of Gram-negative bacteria and *Staphylococcus aureus*, being distinct from healthy individuals, who present higher levels of Gram-positive bacteria such as *Streptococcus viridans* [60, 61].

Chlorhexidine has been considered to be the ‘gold standard’ for oral hygiene in an ICU. However, it has also been associated with bacterial resistance. Due to the known PDT effects against different microorganisms and the absence of a microorganism resistance, this appears to be an interesting possibility. Some studies have shown that an MB vehicle can affect the efficacy of PDT [45, 46, 49]. Due to these facts, the study design will include the treatment that is considered to be the gold standard, together with PDT comprising two different vehicles for MB.

The Hospital Assembly of Mandaqui is a public hospital in the city of São Paulo, Brazil. The ICU receives some adult patients suffering from polytrauma. However, most of the patients are older people and are edentulous individuals. Due to the known differences between oral microbiota and edentulous and *dentate* individuals [4, 62], the panorama of inpatients in the hospital where this study is going to be conducted will only include those edentulous individuals in this protocol. Since different pathogens can co-exist in the same patient, the use of specific selective media to grow and count colonies would be expensive and time consuming. Therefore, our proposal is to perform the quantification by PCR when using a universal (16S) primer which can identify the total bacteria count. However, bacterial quantification by PCR can be puzzling, since the DNA of both live and dead bacteria would be measured together, generating conflicting results [63]. Bacterial quantification by PCR when using PMA achieves the selective detection of living bacteria by an easier method and is an innovation in terms of the analysis of patient samples in phase 1 of this study. PMA can be used as a viability marker as it is a chemical that crosses the dead cell membrane and when it is exposed to light, it intercalates the DNA strands, preventing their amplification. PMA allows for good results for different bacterial species in different types of samples [50, 64–68].

When considering microorganism resistance to many different antimicrobials [69], PDT appears to be an interesting tool, since it can be considered to be effective, independent of microorganism resistance. In addition, by using a low-cost photosensitiser and an illumination device, it becomes attractive for use in the Brazilian public health system. In this sense, MB is a low-cost photosensitiser and it has been widely studied in order to reduce pathogenic microorganisms in oral microbiota [16, 70–72]. However, this is the first trial to evaluate MB in a mouthwash formula that can increase its effectiveness due to the control of MB aggregation.

### Trial status

This is an ongoing trial; thus, the recruiting of participants has not yet started. Patient recruitment has been proposed for May 2018 (phase 1) and for May 2019 (phase 2). Since the approval of this protocol, some modifications have been made. The treatment groups have been reduced to three. The researchers’ role in this research has been revised in order to produce a double-blind trial. Additionally, the number of participants per group has also been recalculated. All of these changes will be duly updated in the REBEC Registry after the publication of this protocol.

## Additional file

Additional file 1: SPIRIT 2013 Checklist: recommended items to address in a clinical trial protocol and related documents, and pages where the items were addressed. (DOC 124 kb)

## Abbreviations

ICU: Intensive care unit
MB: Methylene blue
PDT: Photodynamic therapy
PMA: Propidium monoazide
VAP: Ventilator-associated pneumonia
